# Renal Auto-Transplantation for Loin Pain Hematuria Syndrome Using a Multidisciplinary Team Model: Intermediate-Term Results

**DOI:** 10.7759/cureus.12379

**Published:** 2020-12-30

**Authors:** Jeffrey Campsen, Gilbert Pan, Keith Quencer, Chong Zhang, Angela Presson, Blake Hamilton

**Affiliations:** 1 Department of Surgery, Division of Transplantation and Advanced Hepatobiliary Surgery, University of Utah School of Medicine/Huntsman Cancer Institute, Salt Lake City, USA; 2 Department of Radiology and Imaging Sciences, Division of Interventional Radiology, University of Utah School of Medicine, Salt Lake City, USA; 3 Department of Medicine, Division of Epidemiology, University of Utah School of Medicine, Salt Lake City, USA; 4 Department of Urology, University of Utah School of Medicine, Salt Lake City, USA

**Keywords:** laparoscopy, transplantation, urology, loin pain hematuria syndrome, renal auto-transplant

## Abstract

Background

Patients with loin pain hematuria syndrome (LPHS) can find relief via multiple modalities, few provide long-term pain control like renal auto-transplantation (RAT). This study evaluates the intermediate effectiveness of the RAT procedure’s ability to achieve long-term pain control and quality of life improvement.

Methods

All patients with suspected LPHS were seen by a multi-disciplinary team (MDT) composed of urologists, interventional radiologists, and transplant surgeons. Clinical history and physical exam, lab values, imaging findings, and response to renal hilar block (RHB) were used to determine LPHS and candidacy for potential RAT. Preoperative, one-year, three-year, and five-year postoperative pain assessment scores and quality of life surveys were administered to each LPHS and potential RAT patient.

Results

Eighty-four LPHS patients were referred for the evaluation of and consultation for the option of RAT. Sixty-four of these patients underwent RHB of which 60 (93.8%) had a positive response, defined as a temporary reduction of pain score by >50%. Forty-six of the 60 patients who responded favorably proceeded to RAT. At the one-year follow-up, there was a 75% reduction in pain with 88.9% of patients experiencing a 50% reduction in pain. At one year, the mean Beck Depression Inventory (BDI) decreased by 65.4%, from an average of 23.7 to 8.2. Similarly, at three years (n = 5) and five years (n = 3), the mean pain scores were 2 and 1.

Conclusions

The MDT evaluation of potential LPHS patients with our protocol and treatment results in an improvement in pain and depression scores in these selected patients.

## Introduction

Loin pain hematuria syndrome (LPHS) was first described in 1967 as otherwise unexplained severe flank pain with gross or microscopic hematuria [1]. Many patients have a history of urinary obstruction [2]; it is theorized that this initial insult creates a cascade of chronic pain that can persist after the obstruction is resolved [2-5]. Medical therapies, such as anticoagulation, beta-blockers, angiotensin inhibitors, and chronic pain control, have been proposed and are largely ineffective [6].

Surgical or other procedure-based therapies include percutaneous regional nerve block, surgical sympathectomy, renal capsulotomy, vascular pedicle denervation, ureterolysis, nephrectomy, and renal auto-transplantation (RAT) [2,7-9]. Previous reports describe RAT as encouraging therapy for LPHS, but with widely variable pain relief ranging between 25% and 88% [10-14].

One reason for the treatment failures of LPHS is the difficulty in establishing the correct diagnosis [5]. We propose that a multidisciplinary team (MDT) evaluation of patients with suspected LPHS is the key to its successful diagnosis and treatment. This evaluation must first include a urological evaluation to eliminate other potential causes of flank pain and hematuria, and subsequent referral to interventional radiology (IR) for percutaneous renal hilar blockade (RHB), as we have previously reported [15]. Evaluation by a transplant surgeon is then performed to determine if a RAT is surgically appropriate.

In this paper, we report our intermediate-term results for patients in whom this standardized MDT evaluation was performed, followed by RAT.

## Materials and methods

We defined LPHS as chronic (> 6 months), unbearable flank pain with or without hematuria in the absence of infection, nephrolithiasis, or upper tract obstruction. An MDT, consisting of a urologist, interventional radiologist, and transplant surgeon, was created to evaluate and treat patients with suspected LPHS. All candidate patients, of all genders, went through the same, standardized evaluation protocol. They were first evaluated by our senior urologist to rule out other causes of flank pain and hematuria. If the urologic evaluation was consistent with probable LPHS, patients were then referred to interventional radiology for an RHB.

Prior to RHB, the interventional radiologist graded and recorded pain reported by the patient using a standard visual analog scale (VAS), where 10 is the worst pain and 0 is no pain. Patients were positioned prone, and the renal artery was identified by non-contrast computed tomography (CT). A 21 G needle was advanced adjacent to the ipsilateral renal artery. A 50/50 mixture of 2% lidocaine and 0.5% bupivacaine was administered with a total volume of 20 mL [15]. A post-procedure pain assessment was then performed. Pain reduction by >50% was considered a positive RHB, and patients were then referred to the transplant surgeon for evaluation for RAT. Evaluation for RAT then included CT angiography to assess the renal anatomy for the feasibility of a transplant procedure.

Prior to surgery, baseline quality of life and pain assessment geared towards LPHS patients developed by our MDT was filled out. Patients also completed the Medical Outcomes Study Questionnaire Form 36 Health Survey (SF-36) [16] and the Beck Depression Index (BDI) [17]. These same surveys were then filled out at one, three, and five years after RAT.

Pain score, BDI, and QOL of 50 patients were summarized before and after surgery. Pain score and BDI were summarized as mean (standard deviation (SD)), median (interquartile range (IQR)), and range. QOL was summarized as frequency and percentage at each level (ordered from not acceptable to very good). The exact Wilcoxon signed-rank test was used to compare scores before and after surgery when both were available where QOL, an ordered categorical variable with five levels, was treated as a numerical.

## Results

Between 2013 and 2020, we evaluated 84 patients for LPHS through a near equal mixture of urology and transplant referrals using our standardized MDT methodology. Of these, 64 patients qualified for an RHB after initial evaluation by our urologist. The mean duration of chronic pain prior to RAT was 5.1 ± 7.4 years. Sixty patients had >50% reduction in pain scores following the RHB. The mean patient age was 32.3 years, and 73% of the patients were female (Table 1). Blocks were performed on the right side in 63% of patients. No complications beyond Clavien-Dindo Class I were encountered from the hilar block. A single Class I complication was reported with transient inguinal pain caused by irritation of the genitofemoral nerve. One patient with a horseshoe kidney underwent a targeted block of two renal arteries.

**Table 1:** Patient demographics and endpoints The operative and postoperative information of patients are reported for the 46 patients who underwent RAT. BMI: body mass index; SD: standard deviation; RAT: renal auto-transplantation

Variable	N = 50
Age (y), mean (SD)	33 (11.2)
Ethnicity no. (%)	
Caucasian	49 (98)
Hispanic	1 (2)
BMI (SD)	28.4 (7.1)
Sex (F) (%)	74%
Operative time (min), mean (SD)*	280.5 (55)
Length of stay (d), mean (SD)*	6.5 (1.8)

Of the 64 patients who underwent RHB, 60 (93.8%) had a >50% reduction in pain; this is considered a positive response. For patients who responded to RHB (>50% reduction in pain), there was an overall decrease in pain by 86% (6.6 ± 1.7 down to 0.9 ± 1.2).

Of the 60 patients with a positive response to RHB, 10 (17%) did not undergo RAT; four were denied by insurance, four patients chose alternative therapies (simple nephrectomy (3), intrathecal pump (1)), and two were not candidates for auto-transplant (horseshoe kidney (1), solitary kidney in a morbidly obese patient with three renal arteries (1)).

Of the 50 remaining patients, 46 underwent RAT and the other four are awaiting their scheduled surgery. Of the 46 patients who underwent RAT, 16 patients (36%) were previously diagnosed with depression and 28 (57%) were taking pain medications chronically. The pain was described as burning by 13 (29%), aching by 27 (60%), stabbing by 39 (87%), and/or dull by 21 (46%). There were no patient deaths or graft loss for all 46 patients that underwent a RAT. The donor nephrectomy portion of the surgery was performed laparoscopically in the standard fashion. Two patients were converted to open nephrectomy because of adhesions from prior surgery. After preparation on a back table, the kidneys were then placed into the patient through a standard lower quadrant transplant incision. Mean operative time was 281 ± 51 minutes.

**Figure 1:**
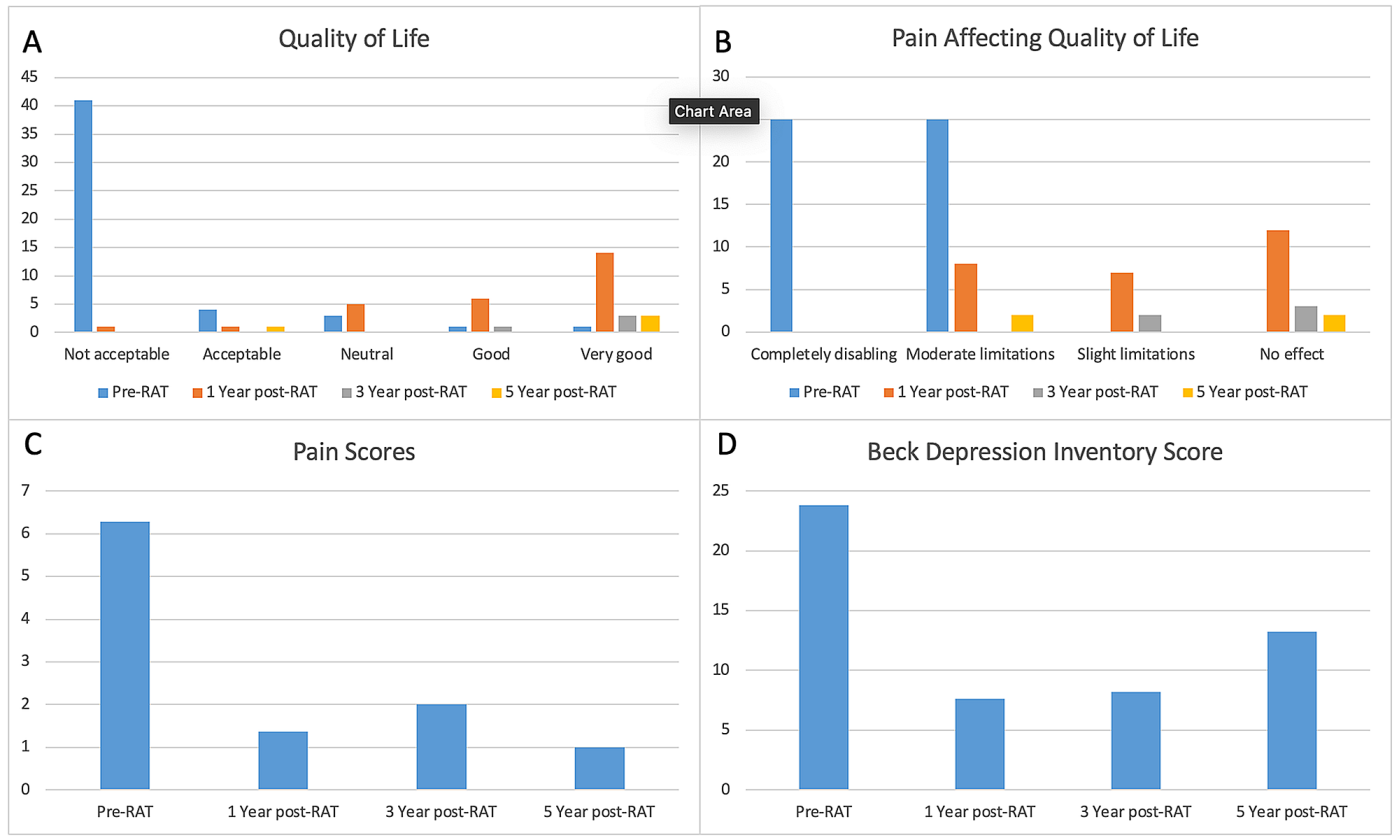
Shifts in quality of life, pain affecting quality of life, and Beck Depression Inventory scores

Of the 46 patients that received RAT, 27 (59.6%) patients have at least one year of follow-up after surgery. Nine patients were lost to follow-up and did not complete the one-year questionnaire. One-year pain assessment was 1.6 ± 2.5 with a mean pain score decrease of 75%. All 27 patients had a sustained improvement in their pain with 85% having a >50% improvement. Mean BDI score (on a scale of 0-63) one year after RAT decreased from 23.7 preop (moderate depression) to 8.2 postop (minimal depression). There were significant shifts in the quality of life, pain affecting the quality of life, and BDI scores (Figure 1). Seventy percent of patients said that RAT was “very successful” in dealing with symptoms and 93% said they “would undergo the procedure again.” Ninety-three percent of patients said they “would recommend the procedure.” Pain one year after RAT was described as non-existent in 15 (57%), aching in 10 (39%), and dull in 6 (21%). Early statistics show continued success in patients with respect to pain, BDI, and QOL for patients at 1 year (Table 2). For all three measures, scores after surgery were significantly lower than before surgery (p<0.001).

**Table 2:** Pain score, Beck Depression Inventory (BDI) score, and quality of life (QOL) pre and post-transplantation at one year (N=50) *Missing values: Pre-op: Pain=3, BDI=8, QOL=3. Post-op: Pain=23, BDI=23, QOL=24*

Variable	Pre	Post	P-value	#pairs
Pain: Mean (SD)	6.4 (2.2)	1.6 (2.5)	<0.001	26
Median (IQR)	7.0 (5.0, 8.0)	0.0 (0.0, 1.5)	-	-
Range	(1.0, 10.0)	(0.0, 8.0)	-	-
BDI: Mean (SD)	23.7 (13.0)	8.2 (9.9)		20
Median (IQR)	23.0 (12.2, 30.8)	3.0 (0.5, 13.5)	-	-
Range	(1.0, 55.0)	(0.0, 33.0)	-	-
QOL: Not acceptable	39 (83%)	1 (3.8%)	<0.001	25
Acceptable	4 (8.5%)	5 (19.2%)	-	-
Neutral	3 (6.4%)	5 (19.2%)	-	-
Good	1 (2.1%)	1 (3.8%)	-	-
Very good	0 (0%)	14 (53.8%)	-	-

Patients reported an average pain assessment score of 2 at three years and 1 at 5 years. Mean BDI score remained low: 8.2 at three years and 13.3 at five years. Of the patients evaluated at three and five years, all would recommend RAT to other patients experiencing similar symptoms. Eighty-eight point nine percent (88.9%) of patients would undergo another RAT if similar symptoms arise with the other one patient opting for ‘maybe.’

## Discussion

Chronic flank pain, often described as loin pain hematuria syndrome, remains an elusive diagnosis that has proven difficult to understand and difficult to treat. When we began our protocol seven years ago, we were motivated by the remarkably dismal outcome of treatments for patients with LPHS. Medical management seemed to be relatively ineffective and carried a tremendous burden since opioids were frequently a staple of those regimens-in an era when we, as a medical community, were trying to decrease our patients’ usage of opioids. Percutaneous ablative techniques were widely disparate, inconsistently applied, and often yielded only transient improvement. Surgical solutions included nephrectomy-often the untimely removal of a functioning kidney in a young patient-and auto-transplant, which had a wide range of success rates yielding a predicted outcome that was often no better than a flip of a coin.

We believed that by the creation of a standardized protocol, employed consistently by a multidisciplinary team, we could both predict and achieve durable results in a greater portion of these patients. We have now demonstrated the success of that program by reporting consistently excellent results at one year and showing a durable trend of success at three and five years.

Our experience now demonstrates that successful treatment for LPHS relies on appropriate diagnosis and the predictive ability of our standardized protocol performed by a multidisciplinary team of clinicians. Our patients have all been previously evaluated by urologists/nephrologists who then refer the patients to our center for LPHS assessment. We report successful screening and triage of patients to RAT through an MDT approach developed at our tertiary referral center. Clinical evaluation by an experienced urologist focuses on eliminating alternative causes of pain and hematuria and identifying appropriate clinical symptoms and history for LPHS. RHB by the interventional radiologist is a critical step in the work-up as it temporarily anesthetizes the renal nerves, predicting the success of RAT.

Prior reports have shown a 25% and a 88% rate of success utilizing RAT for pain control in LPHS, which is an unacceptably low rate of success considering the magnitude of surgery [10-11]. The latter study also reported graft loss of two of the transplanted kidneys [10]. Using our MDT model, 100% of our RAT patients experienced a decrease in their chronic flank pain at one year. A majority of patients (53.6%) had complete resolution of pain while the remaining patients (46.4%) with some continued pain had a mean decrease in pain scores from 6.4 to 1.6 (75% decrease).

In addition to the reduction in pain scores, our patients’ mental health and quality of life were also improved. Not surprisingly, chronic pain is frequently accompanied by an adverse impact on mental health and overall quality of life. In our cohort, patients went from being moderately depressed (mean score of 23.7) preoperatively to minimally depressed at one year (mean score 8.2). Likewise, there was an observed improvement in the quality of life from “not acceptable” to “very good.” Each of these improved measures is consistent with the overall improvement of these patients’ well-being.

When we examine the three- and five-year data, the trends appear to hold up, suggesting a durable response. Patients reported a BDI score of 8.2 at three years and 13.2 at five years. An increase in BDI score is noted in the five-year patient population; however, according to surveyed patients, the increased scores are attributed to disease processes not associated with loin pain. The observed sustained improvement in quality of life affirms the aforementioned statement with a majority of three- and five-year patients reporting ‘very good.’

Although success rates for RAT in the existing literature are low, it is unclear from these reports how the diagnosis of LPHS was made. MDT with protocolized evaluation can help appropriately select patients for RAT with prediction for successful outcomes. A transplant program that has a high-volume live donor kidney transplant program with excellent results as defined by the Scientific Registry of Transplant Recipients (SRTR) [18] is another essential component of MDT’s success.

A limitation of this study is the smaller number of patients with three- and five-year follow-up data. Early in the study period, we treated fewer patients and had a greater number of patients rejected for treatment by insurers. In addition, there were patients who were lost to follow-up, in part because of the large geographical referral patterns, as well as early lapses in protocol or patients who opted out of the study.

## Conclusions

In conclusion, RAT should be considered an excellent treatment option for appropriate patients after standardized evaluation by a multidisciplinary team. This study confirms that when MDT is used with an RHB as part of the workup protocol, RAT is highly successful with a durable reduction in pain and depression scores and patients’ quality of life.
